# Trends of arthroscopy publications in PubMed and Scopus

**DOI:** 10.1186/s43019-021-00096-1

**Published:** 2021-04-29

**Authors:** Srinivas B. S. Kambhampati, Abhishek Vaish, Raju Vaishya, Mohit Kumar Patralekh

**Affiliations:** 1 Sri Dhaatri Orthopaedic, Maternity & Gynaecology Center, SKDGOC, Vijayawada, Andhra Pradesh India; 2 Indian Journal of Orthopaedics, New Delhi, India; 3 Journal of Clinical Orthopaedics and Trauma, New Delhi, India; 4 Department of Orthopedics, Indraprastha Apollo Hospitals, New Delhi, India; 5 Indian Cartilage Society, Ahmedabad, India; 6 Safdarjung Hospital and Vardhman Mahavir Medical College, New Delhi, India

## Abstract

**Purpose:**

Arthroscopy is an established sub-speciality in orthopaedics. With advancing technology, instrumentation and implants, this sub-speciality has seen an explosion of knowledge and techniques since its inception. The indications for arthroscopic management are increasing and, hence, the number of publications on this topic. There has been no study looking into the bibliometrics of all publications within this speciality. The purpose of this study was to look into the trends of published articles on arthroscopy from PubMed and Scopus including studying their citation numbers.

**Materials and methods:**

We set out to look into the number of publications from the earliest up to 2019 and their trends and citation numbers in PubMed and Scopus. We also performed a VOS viewer analysis of MeSH terms and titles of publications to look at research trends over time.

**Results:**

There were 41,149 articles published on PubMed since 1955 and 50,373 articles on Scopus since 1939. The total number of citations were 912,630 for 38,338 cited articles. With 2864 publications in 2019, there was a more than four-fold increase from the number published in the year 2000. The knee joint was the most frequently published joint with an increasing trend in hip arthroscopy. Cohort studies were the most common with 13,180 articles followed by Reviews with 5746 articles. The top 10 authors, universities and journals were listed along with citation numbers. We analysed the trends of publications for each joint and compared them. Yearly citations have progressively increased to reach a maximum of 45,407 in 2007. Arthroscopy was the most published and cited journal on this topic. *The Journal of Bone and Joint Surgery* (*JBJS*) (Am) had the most citations per article. The USA and Hospital for Special Surgery, New York were the most published country and university, respectively.

**Conclusions:**

There is a healthy growth of publications on the subject of arthroscopy with a steep increase in the number of publications and citations in recent years. VOS Viewer analysis showed an evolution of research and practice in the field of arthroscopy. Recommendations were made for databases and search engines to improve on the search and analysis of such studies in the future.

**Level of evidence:**

4

**Supplementary Information:**

The online version contains supplementary material available at 10.1186/s43019-021-00096-1.

## Clinical relevance

This article gives an idea of the magnitude of research and publications within the field of arthroscopy. In addition, trends indicate when, location, the fields where most active research work is happening and who has been doing work on which of the sub-specialities. This study could also act as a reference for comparing future trends.

## Introduction

Arthroscopy has come a long way since it was first reported in 1937. Having started with one journal in 1985, this sub-speciality in orthopaedics can now boast of publications in several high-quality journals with good impact factors and an increasing number of publications every year. As the indications for arthroscopy increase, including the development of newer technologies and the management of more joints and extra-articular tissues using arthroscopy, coupled with faster recovery of these patients compared to conventional surgery and more surgeons taking up this form of surgery, the publications on arthroscopy are expected to increase in the future.

There are multiple articles on publication trends in the literature on arthroscopy-related topics [1–3] and the usefulness of these trends has been highlighted in these. The main limiting factor in doing a similar paper on a wider topic, such as arthroscopy, is the number of articles that need to be analysed. As the number of these increases, the inaccuracies increase and, hence, accurate reproducibility of values in the database decreases, but the trends are not affected [1]. The main challenge in such analyses is handling the large number of articles which would require computers with good processing power and software with a good handling record. For such large numbers of publications, we believe that the trends would be similar in data derived from most search engines. Hence, we used PubMed and Scopus to complement each other to fill gaps in the information. We analysed the citations on this broad topic in PubMed and Scopus to look into the numbers published by authors, universities and countries along with their citation numbers and also based on speciality and study types to see the trends. Further analysis of the frequency numbers and links between authors and MeSH terms was proposed using the VOS Viewer.

A bibliometric study on the broader speciality of arthroscopy would help us identify trends of developments within this speciality, the extent of academic interest within the sub-specialities and various universities and countries and identify sources of information and training, such as journals, authors and universities. The purpose of our study was to identify and analyse this information.

## Materials and methods

The search strategy used was ‘Arthroscop*’ with a filter between the earliest entry to 2019. We included all articles which resulted from this output. The same search strategy was used both in PubMed and Scopus on 1 April 2020. We used Scopus mainly because some data, such as citation counts, university and country of publication are not given in the PubMed database as an output. For this information, we looked at the Scopus database. Data and text-mining, analysis and visualising using VOS Viewer (version 1.6.16) was done on PubMed articles to look into the links and numbers for authors and terms within the titles of the articles to show frequencies and trends over time.

Output was collected from each database and analysed and presented using Microsoft Excel 365. We looked at the data for first authors, top 10 authors in any position, year-wise publications based on sub-speciality, and types of studies, yearly citations of all publications, as well as for the top 10 authors, universities, journals and countries and analysed their citations. We mined the terms from the titles as well as MeSH terms in VOS Viewer and extracted the most prominent terms in every 5-year interval from the year 2000 for MeSH terms and the terms in titles of publications to look at the most prominent research trends over time and presented this information in a table format.

## Results

### PubMed

There were 41,149 articles recorded and published from 1955 to 2019. On PubMed Central, 5869 of these are available as free full text. Publications crossed 500 per year in 1993, 1000 per year in 2006 and, 2000 per year in 2013. In 2019, the number of publications were 2864 which is more than a four-fold increase in the number for the year 2000.

### Top 10 first authors

There were 19,982 and 28,186 unique first authors in PubMed and Scopus, respectively. The number of publications by the first authors were similar in both groups. The order of the authors differed. Scopus did not show up two (Eriksson E and Burkhart SS) in its top 10 authors.

The top 10 authors in all positions in PubMed and Scopus numbers were similar in both databases (Chart 1 and Table 1). The order was slightly different in Scopus. Only the last author in the groups (blank cells) did not belong to the top 10 in each group. The top two values in each column have been highlighted.

**Table 1:** Top 10 first authors in PubMed and Scopus with their number of publications

First author	PubMed	Scopus
Lubowitz JH	232	234
Lui TH	177	134
Kim SJ	127	101
Barber FA	115	69
Eriksson E	76	30
Jerosch J	76	61
Ahn JH	74	68
Burkhart SS	68	44
Byrd JW	57	48
Longo UG	57	48

If the top 10 authors were arranged according to total number of citations, Cole B had the most citations with 9134 followed by Fu FH with 8918 and Kim S with 8520 (Table 1). The top 10 authors by Scopus (Chart 2) does not show the five following authors in the top 10 – Kim S, Kim J, Lee S, Wang J and Lee J. It is possible that there are multiple authors with those surnames and initials as they are common surnames in South Korea and China. Another possibility is that the name is not expressed consistently leaving out initials where multiple initials are present. If the Scopus list was analysed according to author IDs, these erroneous lists would be eliminated. The following Chart 2 gives the actual top 10 authors in Scopus on their website. There is slight discrepancy between the numbers given in the website and the database.

We also looked at the authors who had the most citations per paper. The most cited paper was by Brittberg M (Chart 1) whose ratio of citations per paper was 398.86. One paper published by Brittberg M in 1994 had a total citation count of 4100 [4]. This is the most cited article in the literature for this search. The next most cited article was by Zhang W [5] in 2008 with 1740 citations.

The graph (Chart 3) shows publications with the given sub-speciality keywords in the titles of articles. Most publications were seen for the knee (cell filled with green in Table 2) followed by shoulder (cell filled with orange in Table 2). Although the number of publications in the second place were for the shoulder, it can be seen from the graph (Chart 3) that in the last few years, the number of publications for the hip have exceeded those for the shoulder. Table 2 gives the year the first publication appeared on PubMed and the number of publications associated with that joint. It is important to remove the additional articles seen in the output while searching for hip-related citations. ‘Relationship’ is a frequent word which contained ‘hip’, and this can be picked up during a search for hip-related articles. If not excluded, it could give a falsely high number for this category.

**Table 2:**
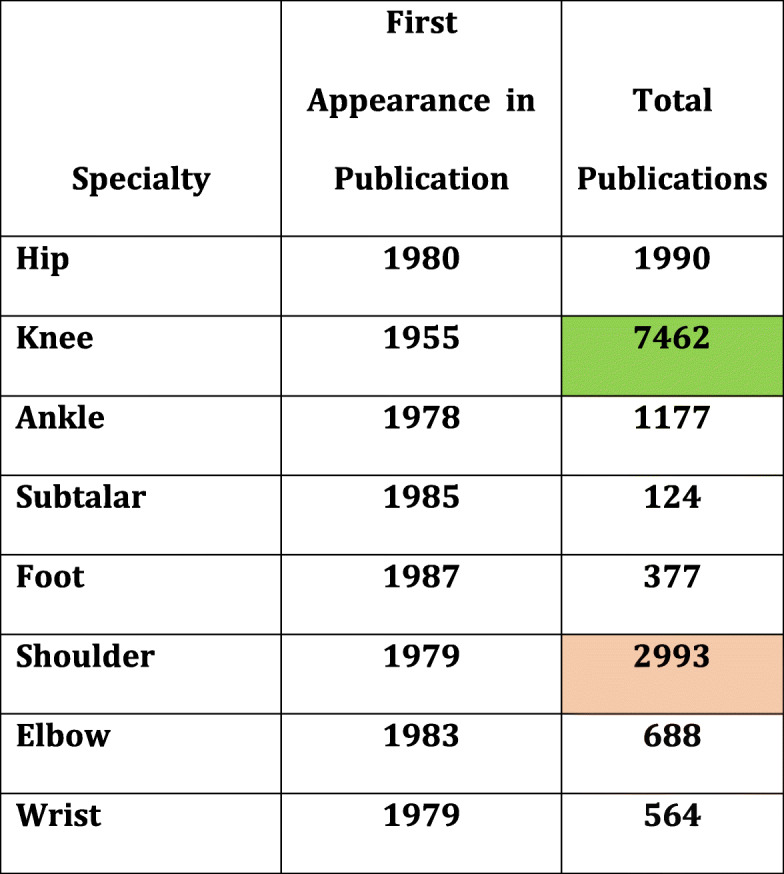
Publication dates of each sub-speciality first appearing and total numbers (data taken from titles)

Chart 4 shows the number of studies distributed by the type of study. Cohort studies were the most common at 32% followed by Reviews. Meta-analyses were the least common at 1.63% only.

### Scopus

The search in Scopus gave an output of 50,373 articles from 1939 to 2019. The total number of cited articles were 38,338 and the total number of citations across all years for all publications were 912,630. The average yearly number of cited papers is 649.8 and the average number of citations per year is 16,296.96. Until 1971, the sum of the number of yearly citations were less than 100. The maximum number of yearly citations was seen in the year 2007 (Chart 5) and the maximum number of cited papers were seen in the year 2016. It can be seen from the chart that the shape of the graph showing the number of publications leads about 8–9 years ahead of the citations graph. The number of publications have been less over the last 3 years, having reached a peak of 2567 in the year 2016.

Graph (Chart 6) shows citation numbers of each of the top 10 authors year by year. The graph and the lines are busy, but the point of the graph is to show that citation numbers of almost all authors began to increase after the year 2000 and most of the peaks are seen in the first half of the last decade. This is to indicate the possibility of impact of the Information Technology revolution beginning from the year 2000 (PubMed went online in 1997) and also that there is a lag of a few years before the top authors’ citations peak in terms of citations of articles.

Among the top 10 countries publishing on this topic, the USA has published the greatest number of articles (Chart 7) with 18,118 (35.97% of total) followed by Germany with 4462 (8.86%).

The Hospital for Special Surgery was the university (Chart 8) with the most publications on this topic with 1028 articles. Among the top 10 universities publishing on this topic, eight belong to the USA. The other two universities belonged to Germany and Netherlands at eighth and ninth places with 374 and 338 publications, respectively.

### VOS Viewer

**Fig. 1:**
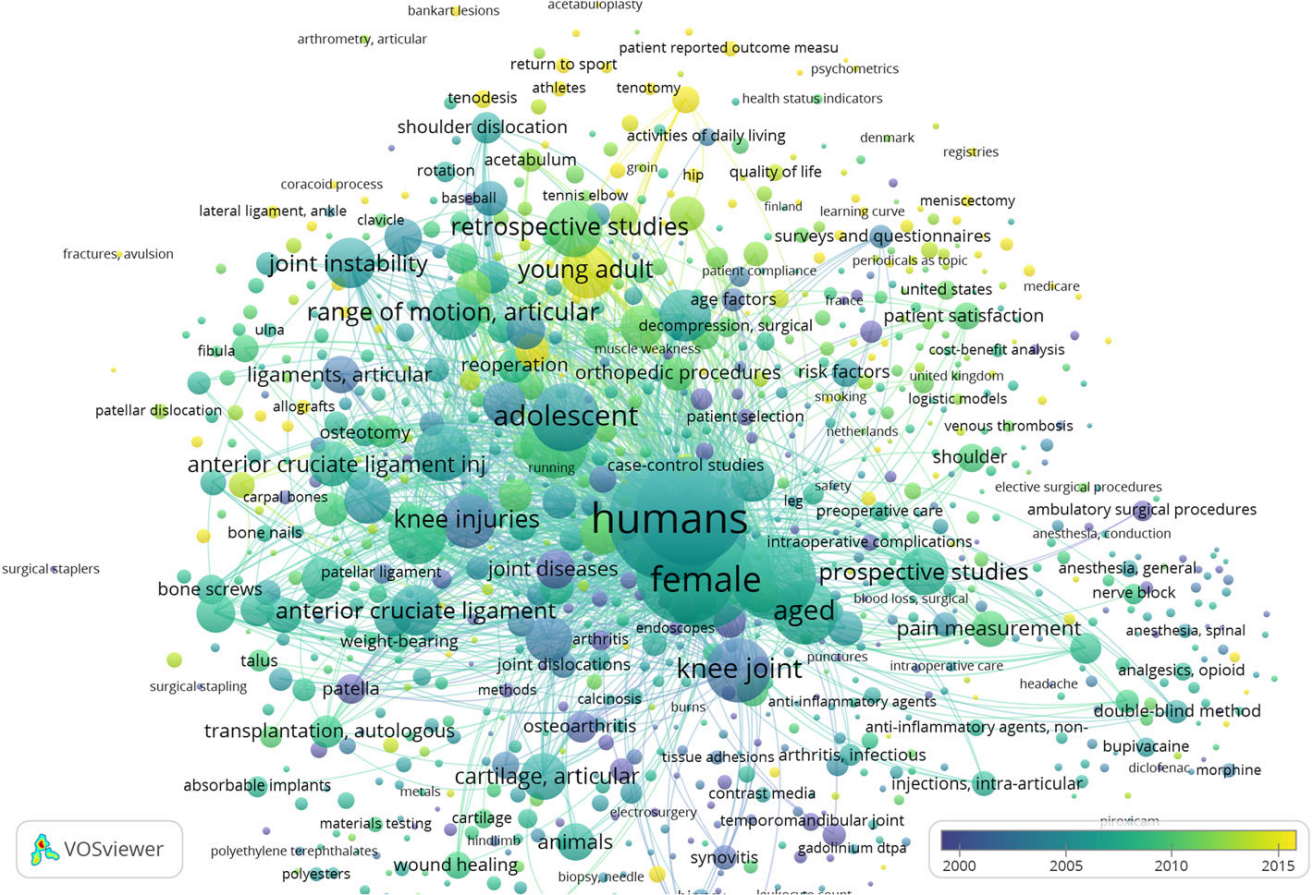
VOS Viewer output for terms from PubMed

VOS Viewer outputs for terms are presented in Fig. 1 and Table 4. VOS Viewer is a text-mining software used for constructing bibliometric networks and it plots the frequency of terms or names of authors and their links with each other and relates them with time from data extracted from PubMed or Scopus searches. The terms mentioned represent the main focus of research during the periods mentioned in Table 4.

MeSH keywords were looked at with a minimum number of occurrences of 20 for each keyword, 1014 keywords were plotted, while 1029 were found to have 24 occurrences or more. These were plotted in the VOS Viewer and major terms listed in the table according to the years that they were seen most.

**Fig. 2:**
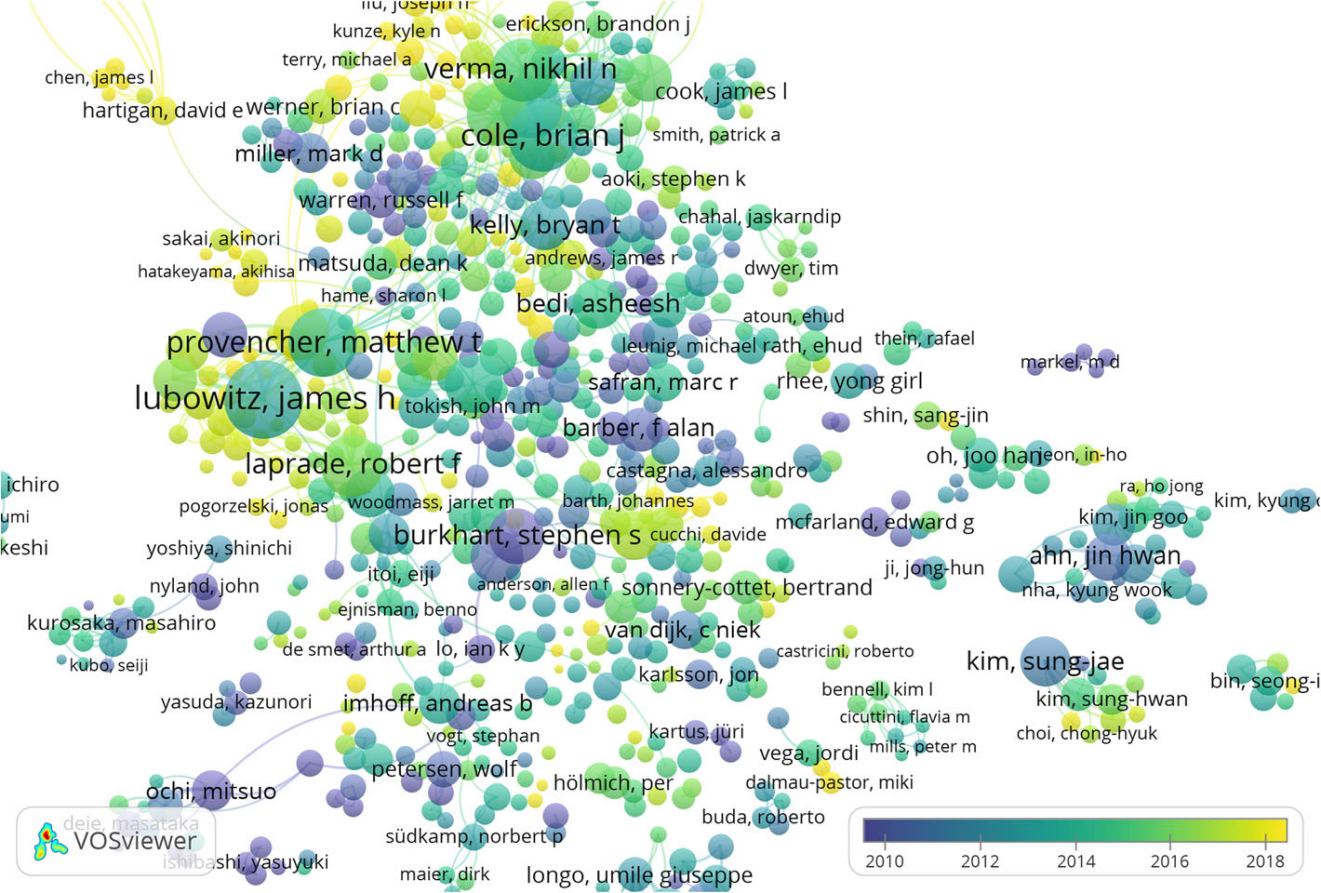
VOS Viewer output for authors from PubMed

VOS Viewer output for authors is shown in Fig. 2. As can be seen, the predominant duration of publications of author is indicated by the colour index given. The number of publications by the author is indicated by the prominence of the name and size of the circle. The bigger the circle, the greater the number of publications. More details on the analysis of VOS Viewer outputs in relation to orthopaedic publications and features are available in previous publications [1, 2] as well as from the creators of the software.

## Discussion

The major databases offering information of citation numbers for articles are Scopus and Web of Science (WOS). PubMed has recently started providing the facility through a different resource but not as an output. Both Scopus and WOS require subscription for using their services. PubMed, on the other hand, is the largest free-for-all science search engine. It does not provide information on citation numbers of articles by default as well as information on universities and countries in a downloadable format. There are some studies in science which analysed bibliography citations using WOS, which has been maintained by Clarivate Analytics since 2016 (previously Intellectual Property and Science business of Thomson Reuters). Scopus, maintained by Elsevier, on the other hand was established in 2004 and is a rapidly growing database. Since both offer similar information on citations, we used Scopus to analyse citations of bibliography on arthroscopy and include information on countries and universities publishing on this topic. We combined PubMed and Scopus for the respective advantages that they provide in extracting the maximum information available on the bibliometrics on arthroscopy. Limitations of doing data analysis from PubMed based on titles of articles have been discussed in previous publications [1, 3]. A publication in 2009 comparing WOS and Scopus concluded that two in three articles are common in both databases with one in three fringe references seen in one or the other [6]. Another publication looking into the most cited papers in arthroscopy in India for 10 years (from 2007 to 2017) found that several papers were missed by WOS [7] that were picked up by Scopus. Advantages and disadvantages of citation analysis were discussed in this previous publication [7]. The decline in citations over the last 5–10 years is due to the known fact that the citation numbers take time to peak.

Established in 1985, *Arthroscopy* was the first dedicated journal publishing literature on arthroscopy. There are now several journals publishing articles exclusively on arthroscopy or in combination with sports injuries. We analysed data on the top 10 journals publishing on arthroscopy.

There were a total of 1597 journals publishing on this topic on PubMed and 3395 journals on Scopus indicating that Scopus includes more journals in its database than PubMed on this topic. The top 10 journals in PubMed and Scopus were almost similar (Table 3). The number of articles that were cited and the total sum of all citations related to a journal could only be calculated from the Scopus data as PubMed does not give the citation numbers for each article.

**Table 3:**
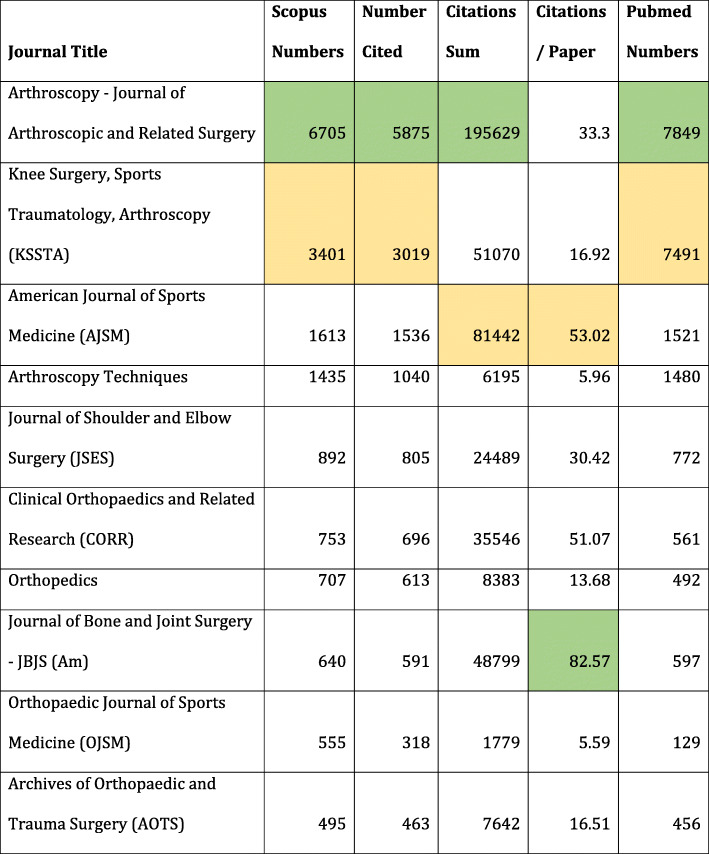
Top 10 journals with Scopus and PubMed numbers, total citations and citations per paper. Maximum value is coloured green followed by orange

*Arthroscopy* was the most published journal followed by *The Knee Surgery, Sports Traumatology, Arthroscopy* (*KSSTA*) and *The American Journal of Sports Medicine* (*AJSM*) in both the databases. They were also the top three most cited journals in that order. The table also gives the total number of articles cited by the journal and the number of citations per paper published by the journal by dividing the total citations with number of articles published on this topic.

The impact factor of a journal is calculated by citations per article published but for all the papers published in a time period by the journal. The ratio given in the Table 3 would be similar but specific for this topic. The maximum all-time topic-specific impact on Arthroscopy was by *The Journal of Bone and Joint Surgery* (*JBJS*) (Am) with 82.57 followed by *AJSM* with 53.02. *JBJS* (Am) and *The Clinical Orthopaedics and Related Research* (*CORR*) are general orthopaedic journals with higher impact factors and, hence, citations of articles in these journals would be expected to be higher than those published in sub-speciality journals like *AJSM* and *KSSTA*. For a sub-speciality journal like *AJSM*, a ratio of 53.02, even higher than *CORR*, is very good.

In the VOS Viewer output, increased occurrences of terms would indicate increased focus on a particular topic. Topics coloured yellow in Fig. 1 are recent ones after 2015. It can be seen from Table 4 that both the terms in the titles as well as MeSH keywords indicate advanced concepts after 2014.

**Table 4:** VOS Viewer output for PubMed for MeSH terms

	Most prominent terms in titles		Most prominent MeSH terms
**> 2014**	Controlled trial, plasma, hip arthroscopy, shoulder arthroscopy, young adult, commentary, tenodesis, medial patellofemoral ligament, Bankart repair, irreparable rotator-cuff tear	**> 2015**	Femoroacetabular impingement, young adult, quality of life, return to sport, athletes, operative time, cost-benefit analysis, registries, patient-reported outcome measures, visual analogue scale
**2010–2014**	Autograft, talus, sports medicine, literature, arthroscopic partial meniscectomy	**2010–2015**	Treatment outcome, rotator-cuff injuries, retrospective studies, hip joint, acetabulum, quality of life, patient satisfaction, magnetic resonance imaging (MRI), hip joint, multivariate analysis, pain measurement
**2006–2010**	Knee arthroscopy, ankle arthroscopy, arthroscopic reconstruction, patellar instability, *remplissage*	**2005–2010**	Adolescent, follow-up studies, shoulder joint, biomechanical phenomena, sensitivity and specificity, autologous transplantation, surveys and questionnaires
**2000–2006**	Tibial tunnel, rheumatoid arthritis, arthroscopic synovectomy, villonodular synovitis	**2000–2005** **moderate weightage**	Knee joint, knee injuries, joint diseases, radiography, menisci, endoscopy, articular cartilage, ligaments, arthritis, loose bodies, ambulatory procedure, patella
		**1990–2000** **low weightage**	Cost-benefit analysis, electromyography, joint prosthesis, gadolinium, patella, osteoarthritis, joint diseases

The focus in recent times appears to be on femoroacetabular impingement, young adult, quality of life, return to sport, athletes, operative time, cost-benefit analysis, registries, patient-reported outcome measures, visual analogue scale indicating advanced concepts in assessment and treatment of sports injuries, although weightage of these terms is low. From the VOS Viewer figure and table, one can see how research and practice in arthroscopy has evolved over time based on the major terms occurring in the given periods of time.

### Limitations

WOS has the advantage of holding data on citations on historical articles dating back to 1900 while Scopus is relatively new with data dating up to the 1970s. But the number of journals and additions per week are far more in Scopus than in WOS. We used Scopus for the advantage with numbers. Major developments in arthroscopy occurred after 1970s and, hence, we felt that a Scopus analysis would suit our study.

One should also keep in mind the drawbacks related to using citation numbers as measures of performance. These include self-citations, omission bias, powerful person bias and institutional or high-impact journal bias and snowball effect.

### Recommendations

We propose inclusion of anatomical location of the joint being studied as well as the type of study in the title or keywords of all manuscripts being submitted to all journals to improve and perform an analysis of the literature easier in future studies for this topic. Keywords should be given in the output data of major search engines to make analysis in bibliometric studies better.

We also propose marking authors by author ID similar to the one in Scopus or ORCID for evaluation of author-related data in the PubMed database. These measures will improve the accuracy and reproducibility of the results obtained by similar studies in the future.

## Conclusions

There were clear trends identified from our study. These include:

- There is a steep increase in the number of cited papers and the number of citations in recent years. Most publications were seen for the knee joint followed by the shoulder and hip. Recent publications per year for hip appear to be more than for shoulder. Cohort studies were the most commonly published followed by reviews. *Arthroscopy* was the most published and cited journal followed by *KSSTA*. *JBJS* (Am) has the most citations per publication followed by *AJSM*.
- Lubowitz JH is the most published author but Brittberg M was the most frequently cited author followed by Cole B, and the USA was the most publishing country. HSS, New York was the most publishing university on this topic.
- VOS Viewer analysis showed the evolution of research and practice in the field of arthroscopy and some of the keywords of evolution were listed. Peak citation numbers for authors were seen to lag 5–6 years before the date of this study.
- We have made recommendations to improve the accuracy and reliability of the search and analysis of such studies in the future.

## Supplementary Information

**Additional file 1: Chart 1.** Top authors in any position. Twelve were selected to include the top 10 from both databases. **Chart 2.** Top 10 authors in Scopus. **Chart 3.** Yearwise publications in each sub-speciality. **Chart 4.** Numbers published according to type in PubMed. **Chart 5.** Yearwise numbers of total yearly citations and number of cited papers from Scopus. **Chart 6.** Yearly citation numbers of top 10 authors in any position in Scopus. **Chart 7.** Number of papers published by Top 10 countries with their percentages. **Chart 8.** Number of publications by top 10 universities.

## Data Availability

The datasets during and/or analysed during the current study available from the corresponding author on reasonable request.
