# Supplementary material for: Trends of arthroscopy publications in PubMed and Scopus
Source: Knee Surg Relat Res. 2021 Apr 29;33:14. doi: 10.1186/s43019-021-00096-1 (PMC8082866; doi:10.1186/s43019-021-00096-1)
Supplement: Supplementary file 1 — Additional file 1: Chart 1. Top authors in any position. Twelve were selected to include the top 10 from both databases. Chart 2. Top 10 authors in Scopus. Chart 3. Yearwise publications in each sub-speciality. Chart 4. Numbers published according to type in PubMed. Chart 5. Yearwise numbers of total yearly citations and number of cited papers from Scopus. Chart 6. Yearly citation numbers of top 10 authors in any position in Scopus. Chart 7. Number of papers published by Top 10 countries with their percentages. Chart 8. Number of publications by top 10 universities. [file 43019_2021_96_MOESM1_ESM.docx]

**Chart1: Top authors in any position. Twelve were selected to include top 10 from both databases. Data Labels are coloured according to the bar colour.**

**Chart 2: Top 10 authors in Scopus**

**Chart 3: Yearwise publications in each subspeciality**


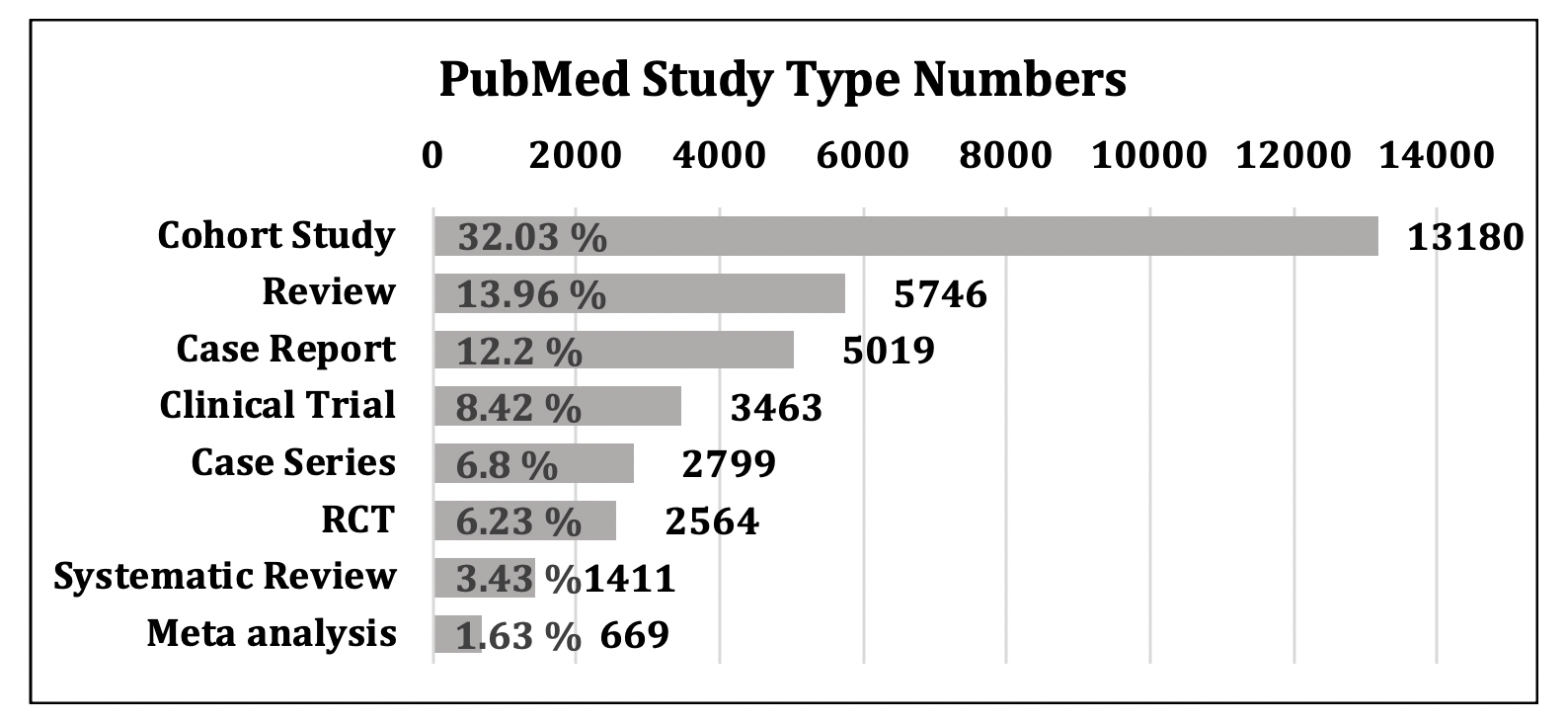


**Chart 4: Numbers published according to type in PubMed**

**Chart 5: Yearwise numbers of Total yearly citations and number of cited papers**

**Chart 6: Yearly citation numbers of top 10 authors in any position in Scopus**


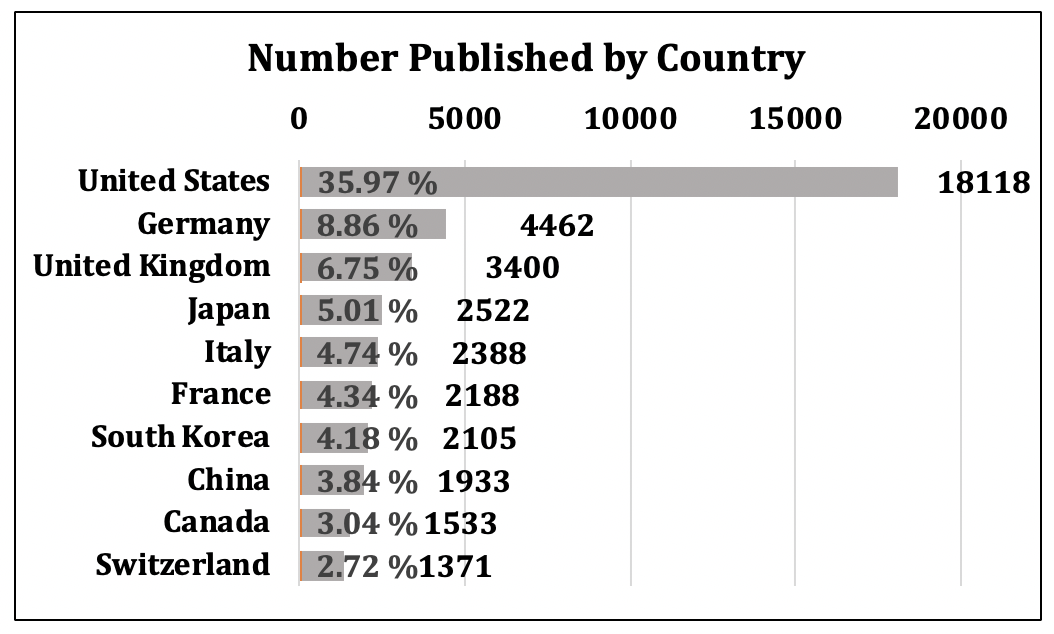


**Chart 7: Number of papers published by country with their percentages**

**Chart 8: Number of publications by University**
